# The Census of Canada

**Published:** 1856-01

**Authors:** 


*The Census of Canada.*—The late census discloses some singular facts. In the United States it has been found—and it is similar in Canada—that under five years of age there are more males, by nearly 50,000, than females; then, between the ages of fifteen and twenty, the females outnumber the males to about the same extent. The tide turns again between the ages of thirty and forty, then the males outnumbered the females, in the year 1850, upwards of 160,000; and last scene of all, at seventy years of age the feminine gender is again in the ascendant. In Canada this remarkable ebbing and flowing of the sexual tide has been found to vary in a similar ratio, differing only in the figures, in proportion to the respective populations of the two countries.

Whether the same result is found in the old countries of Europe the report does not say; but it is not probable. Different circumstances there produce different results upon the population, as the following fact shows:—Of 42,000 families in Britain, each with a husband and wife at its head, it is found that 12,000 have no children at home; 8000 have but one; 7000 two; 5000 three; 4000 four; and so on, heads of families decreasing in numbers as the number of children increases. In opposition to this Canada shows a remarkable difference. Out of 42,000 families there, only about 4000 were without children with them, and so on in proportion between the numbers of families taken in the respective countries, large families being more plentiful in Canada than in Britain. The report accounts for this difference in several ways. One is, that emigrants to the country generally come out at a vigorous productive age, this reason seems valid. Another which he offers does not seem quite so much so; that is, early marriages in Canada. It is scarcely possible that these can prevail to a greater degree in Canada than in Great Britain. It is a well known fact that among the working classes of the three kingdoms early marriages prevail to a ruinous and deplorable extent.

Another singular fact which the report brings before us is the discrepancy in the number of births and deaths between the two provinces. In 1851, the births of Upper Canada were, leaving out the odd numbers, 32,000; while the deaths amounted to 7000, being something less than a fourth part of the births. In Lower Canada, during the same year, the births were in round numbers, 36,000; the deaths 11,000, nearly a third part of the number born, thus showing a heavier mortality in the lower province than in the upper. The report states that epidemics and children's complaints, such as hooping-cough, croup, etc., are much more fatal in Lower than in Upper Canada.

Not the least interesting item in the census is the return of the Indian population, these ancient lords of the forest. The report states their whole number in the two provinces to be 8728; but it says this is probably little over half their number, as it is known many of them live beyond the census limits. In support of this it is stated that Smith, in his work on Canada, enumerated them at 14,000 in 1842.

The most melancholy part of the report is that relating to the deaf and dumb, blind, and lunatic. Here, again, the lower province appears to disadvantage. The number of deaf and dumb in Lower Canada is 865; in the upper provinces it is little more than the half, 478. In Lower Canada there is one to every 1029 of the whole population; in Upper Canada there is one to every 1991 of the population; while the census of the United States shows a proportion even still less, they having one to every 2395 of the whole population.

Of lunatics Lower Canada has 5733, one to every 543 of the population; Upper Canada has 1069, one to every 380 of the whole inhabitants.

The number of inhabitants who had either reached or passed their hundredth year in Canada East, at the taking of the census, was thirty-eight, twenty females and eighteen males; only four out of the whole number were married, but twenty-three were widows and widowers; only eleven out of the whole having lived out their century in single blessedness, and of these eleven only three were males.

The report comprises some very interesting returns going to show the remarkable health of the Canadas. There were over 100 years of age in Upper Canada, fourteen males and nineteen females. Two males were respectively 115 and 120. Two females were each 106 and 114 Nor is the eastern province in any way behind in these instances of longevity, the return showing that there were twenty-two males over 100 years of age, and eighteen females.—*Edinburgh Med. Journal from Edin. News, Aug. 11th.*

